# Ih Current Is Necessary to Maintain Normal Dopamine Fluctuations and Sleep Consolidation in *Drosophila*

**DOI:** 10.1371/journal.pone.0036477

**Published:** 2012-05-04

**Authors:** Alicia Gonzalo-Gomez, Enrique Turiegano, Yolanda León, Isabel Molina, Laura Torroja, Inmaculada Canal

**Affiliations:** Departamento de Biología, Universidad Autónoma de Madrid, Madrid, Spain; University of Chicago, United States of America

## Abstract

HCN channels are becoming pharmacological targets mainly in cardiac diseases. But apart from their well-known role in heart pacemaking, these channels are widely expressed in the nervous system where they contribute to the neuron firing pattern. Consequently, abolishing Ih current might have detrimental consequences in a big repertoire of behavioral traits. Several studies in mammals have identified the Ih current as an important determinant of the firing activity of dopaminergic neurons, and recent evidences link alterations in this current to various dopamine-related disorders. We used the model organism *Drosophila melanogaster* to investigate how lack of Ih current affects dopamine levels and the behavioral consequences in the sleep∶activity pattern. Unlike mammals, in *Drosophila* there is only one gene encoding HCN channels. We generated a deficiency of the *DmIh* core gene region and measured, by HPLC, levels of dopamine. Our data demonstrate daily variations of dopamine in wild-type fly heads. Lack of Ih current dramatically alters dopamine pattern, but different mechanisms seem to operate during light and dark conditions. Behaviorally, *DmIh* mutant flies display alterations in the rest∶activity pattern, and altered circadian rhythms. Our data strongly suggest that Ih current is necessary to prevent dopamine overproduction at dark, while light input allows cycling of dopamine in an Ih current dependent manner. Moreover, lack of Ih current results in behavioral defects that are consistent with altered dopamine levels.

## Introduction

Hyperpolarization-activated cyclic nucleotide-gated channels (HCN) are responsible for the Ih current, which regulates rhythmic electrical activity, contributes to the resting membrane potential, and shapes the input-output curve in excitable cells. The Ih current was originally described as the inward cation current activated at hyperpolarized membrane potentials which substantially contributes to the spontaneous pacemaker activity of the heart sinoatrial cells. Indeed, attempts to ameliorate cardiac rhythm disorders have established HCN channels as promising drug targets [1], [2]. However, recent studies have shown that HCN channels are associated not only with cardiac dysfunction, but also with neurological disorders such as neuropathic pain and epilepsy [2], [3]. Thus, pharmacological manipulation of HCN channels represents a great potential for the development of cures to treat these debilitating diseases.

In mammals, HCN channels are encoded by four genes, HCN1–4, that show distinctive but partially overlapping expression in different tissues and brain regions [4]. In recent years several groups have investigated the phenotypic consequences of knocking out single HCN channel-encoding genes, and gene-specific neural defects have been reported [3], [5]. Nevertheless, how the different HCN genes contribute to diverse neurological dysfunctions is still unclear. Moreover, because the Ih blockers developed thus far show no subtype-specificity for the different HCN genes [6], therapeutic testing of these pharmacological agents will benefit from considering the effects of the total lack of Ih current in different neural outcomes. Taking advantage of the fact that in *Drosophila* there is only one HCN channel encoding gene, *DmIh* [7], we have abolished Ih current by deleting a core region of the channel. This new mutant provides an ideal model to study the possible effects of the lack of the Ih current in the whole organism.

In rodents, dopaminergic neurons display characteristic rhythmic spontaneous firing activity, which is dynamically modulated by multiple afferent inputs. Several studies have identified Ih current as an important determinant of this spontaneous firing rate [8]–[12]. Moreover, many neurotransmitters target HCN channels to modulate the afferent stimuli-dependent activity of dopaminergic cells [13]–[17]. Increasing evidences suggest that HCN channels also play relevant roles in several dopamine-related disorders, such as drug addiction [18], [19], schizophrenia [20], or Parkinson disease [21], [22]. In spite of the growing data on Ih modulation of dopaminergic neuronal function [23], the final consequences of altering this current over the dopamine *in vivo* output have not been reported. Therefore, we analyzed how impairment of Ih current in *DmIh* mutant flies may alter dopamine outcome *in vivo*. *DmIh* gene is broadly expressed in the *Drosophila* brain [24], but precise cell-type localization experiments have not been done. Our results show that indeed *Drosophila* brain dopaminergic cells express *DmIh*. Moreover, we provide the first demonstration of significant circadian variation in levels of dopamine in *Drosophila* head extracts, and show that the daily cycling of dopamine is drastically modified when Ih current is eliminated. Therefore, our data associate Ih current with dopamine signaling also in flies, suggesting a role for DmIh channels in influencing dopamine neurotransmission either in a dopaminergic cell-autonomous manner or at different steps in neuronal communication.

Several experiments and observations demonstrate a central role for dopamine in regulating sleep–wake states. For instance, mice with varying degrees of dopamine depletion show different levels of REM sleep [25], and also in flies dopaminergic signaling is important for the regulation of sleep [26]. Subsequently, *DmIh* mutant flies display alterations in the rest∶activity pattern which correlate with the aberrant dopamine levels. Finally, flies lacking Ih current show altered circadian rhythms, which translate into an arrhythmic behavior or a shorter period in constant darkness conditions.

## Results

### Generation of *DmIh* null mutant

**Figure 1:**
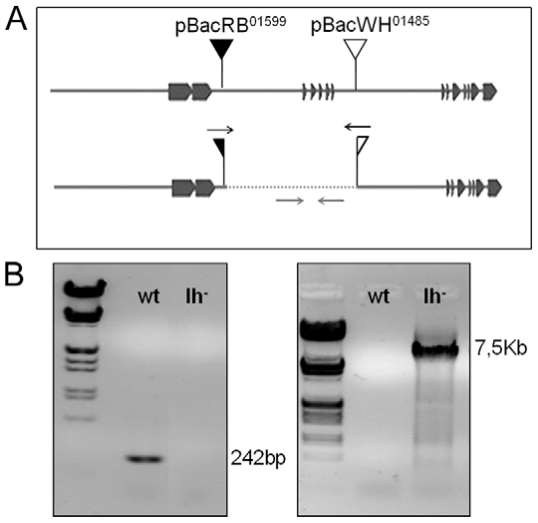
Generation of the *DmIh* null mutant. (A) Diagram of the *DmIh* gene showing the position of the two piggyBac transposon insertion sites. The dotted line at the bottom scheme corresponds to the deleted region upon recombination. Black arrows indicate the position of the primers used to amplify the newly formed piggyBac element (amplicon size 7.5 Kb). Gray arrows indicate the position of the primers inside the deleted region (amplicon size 242 bp). (B) PCR results show lack of amplification of the presumptive deleted region in the *Ih^−^* line, further confirmed with the positive amplification of the newly formed piggyBac element.

In *Drosophila*, HCN channels are encoded by a single gene (*DmIh*; FBgn0028428) that suffers alternative splicing, potentially giving rise to at least 12 isoforms [7], all of which share the S1 to S3 transmembrane domains encoded by exons 7 and 8 (based on Flybase gene annotation). In situ hybridization with a probe containing this common region has revealed pan-neural expression in fly adult brain [24]. Therefore, to create the null mutant, two piggyBac element insertion lines were used to delete the sequence between exons 4 and 12 of *DmIh* by FLP-FRT based recombination [27]. pBac^RB01599^ is inserted upstream of exon 4 and pBac^WH01485^ is inserted downstream of exon 12. Upon heatshock, recombination occurs between these two piggyBac elements, deleting the intervening region and regenerating a complete piggyBac element from half of each of the original piggyBacs (Figure 1A). The deletion was confirmed by PCR using genomic primers inside this region (Figure 1B; amplicon size 242 bp). The recombination event that originated the deletion was further confirmed by PCR, using primers 3′ of the piggyBac elements to amplify across the newly formed element (Figure 1B; amplicon size ∼7.5 kb). The *DmIh* deletion line was backcrossed into a Canton-S (CS) background for five generations for further analysis, and one control (*wt*) and one mutant (*DmIh*) isogenic line was established and used in all experiments. Care was taken to completely eliminate the *w^1118^* mutation to avoid an effect on dopamine levels [28].

### Dopamine shows daily and circadian oscillations in *Drosophila* heads

**Figure 2:**
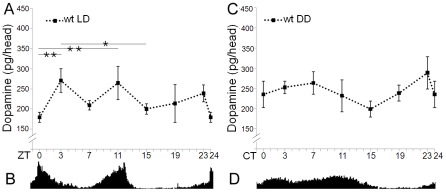
Circadian levels of dopamine in control flies. Dopamine and activity was measured in 6 to 8 days old flies at day 3 or 4 in each condition. Error bars indicate SEM. (A) In LD conditions, control flies show daily cycling of dopamine amount with two peaks at daytime and reduced levels at nighttime. Bars at the top indicate significant difference between two points (*p<0.05, **p<0.01, post hoc Bonferroni test). (B) In this condition the total daily activity plot (n = 32) reveals the typical bimodal pattern of activity with two maximums at the moment of lights on (ZT0) and of lights off (ZT12). (C) When transferred to constant darkness, the cycling of dopamine in control flies is attenuated. (D) Total daily activity plot (n = 47) in DD conditions shows the characteristic sustained activity plateau during the subjective day (CT0-CT12).

In rat, the amount of dopamine fluctuates in a circadian manner, at least in the striatum and nucleus accumbens [29], as do other proteins implicated in dopaminergic transmission, such as the dopamine transporter (DAT), or tyrosine hydroxylase (TH), the rate-limiting enzyme in dopamine biosynthesis [30]. In *Drosophila*, both TH [31], [32] and the dopamine receptor responsiveness [33] appear to be under circadian control, suggesting cyclic variations of dopamine levels, although direct evidence is lacking. To evaluate this possibility, we quantified dopamine in *Drosophila* heads every four hours, with some additional measures when considered necessary, under 12 h light∶dark (LD) conditions (Figure 2A).

Dopamine amount shows a rhythmic behavior, peaking at the beginning and the end of the light period, with lowest levels in the middle of the day. At nighttime dopamine is maintained to a low level, slowly increasing towards the end of the night (Figure 2A). Coincident with lights on, dopamine amount falls to a minimum to regain the cyclic behavior. Cycling of dopamine was further supported by one-way ANOVA, which showed a significant effect of the time of day (ZT) on dopamine levels (F_6,21_ = 5.719; p = 0.001). Although previous indirect cues suggested cycling of dopamine levels in *Drosophila* [31], [32], [34], our data provide the first direct evidence of its daily oscillation.

In rat, the release of dopamine in the striatum also shows daily oscillations in LD conditions [29], [35]. These oscillations disappear under constant dark conditions, suggesting that they are light-dependent [29]. However, in the nucleus accumbens extracellular dopamine displays a circadian rhythm that continues under constant dark conditions [29]. To find out if cycling of dopamine in *Drosophila* heads is influenced by light or under circadian control, we measured dopamine in flies kept in constant darkness (DD).

Flies were entrained for two days in LD prior to being released to DD, and dopamine was determined after three days in this condition. As shown in Figure 2C, dopamine levels do not fluctuate during the subjective day. Instead, high values of dopamine are maintained throughout most of the subjective day, decaying to the lowest levels at the transition between the two subjective periods, and increasing again to reach the highest point at the end of the subjective night. Indeed, one-way ANOVA shows an effect of the time of day (CT) on dopamine levels also in DD (F_6,21_ = 3.670; p = 0.012), although post hoc analysis failed to identify significant differences between any of the dopamine data points.

The different pattern of dopamine oscillation observed in LD and DD suggests that light affects dopamine levels also in fly heads. In addition, the similar pattern of dopamine oscillations during the night period in LD and the subjective night in DD indicates that dopamine levels could be under circadian control during dark periods. To test this hypothesis, we analyzed the effect of circadian time (ZT or CT) and light conditions (LD or DD) on dopamine levels by a two-way ANOVA. The results show that both factors, as well as their interaction, have a significant effect (F_13,42_ = 4.760; p<0.001; for details see Table S1), supporting that circadian changes of dopamine amount are different in LD and DD. Moreover, student t-test analysis showed that the only time points in which dopamine levels differ between LD and DD conditions occur during the day (t_6_ = −3.18; p<0.5 for ZT/CT0 and t_6_ = −3.57; p<0.5 for ZT/CT7), which corroborates a role for light in controlling dopamine levels.

### Lack of Ih current modifies dopamine rhythmicity in LD conditions

**Figure 3:**
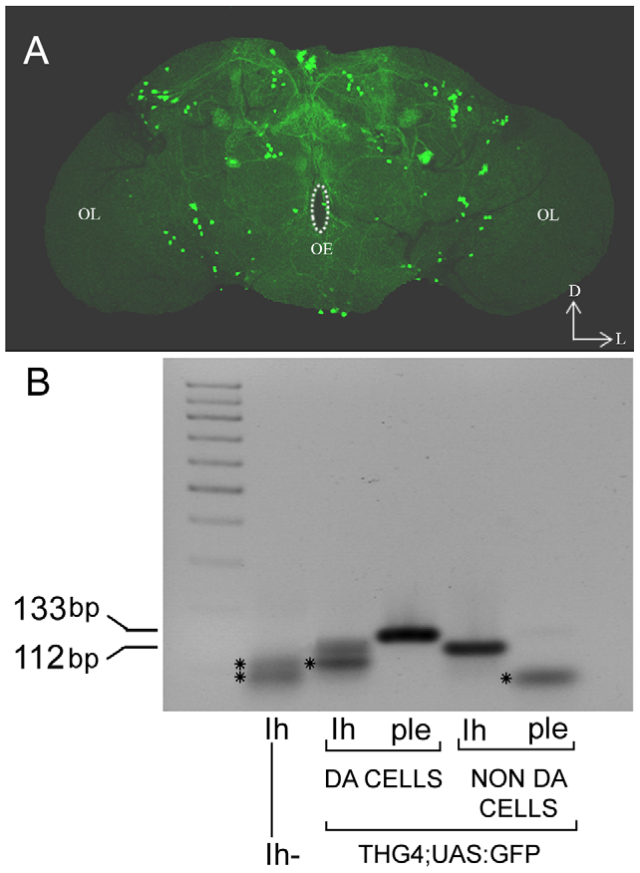
*Ih* gene is expressed in dopaminergic neurons. (A) Confocal projection of adult brain showing dopaminergic neurons labeled with GFP using the Tyrosine Hydroxilase Gal4 driver (*THG4;UAS:GFP*). D, dorsal, L, lateral, OE, oesophagus, OL, optic lobe. (B) RT-PCR amplification of *Ih* (112 bp) and *ple* (133 bp) RNAs from 200 isolated dopaminergic and non-dopaminergic neurons sorted by Fluorescence Activated Cell Sorting (FACS). Image shows amplification of *Ih* RNA in dopaminergic, *ple*-expressing neurons. Failure to PCR-amplify the neuronal *ple* RNA isoform in non-dopaminergic neurons (GFP negative cells) confirms that cells have been correctly sorted. Asterisks in lanes 1, 2, and 5 point to nonspecific bands, which were obtained in the negative controls (*DmIh* mutant brain-RNA extract for *Ih* –lane 1- and GFP-negative cell-RNA extract for *ple* –lane 5-).

A number of electrophysiological experiments, either in mice midbrain slices [8] or in dissociated neurons [9], support the implication of Ih current in the autonomous pacemaker frequency of dopaminergic neurons. Moreover, various neuromodulators have been reported to regulate excitability of dopaminergic neurons through modulation of Ih current [13]–[17]. We reasoned that if the Ih current is responsible for the spontaneous firing of many excitable cells in mouse, lack of this current could dampen or even cancel the rhythmic levels of dopamine in fly, via its effect on intrinsic activity not only of dopaminergic neurons, but also of other neurons that directly or indirectly influence their synaptic inputs. In the fly brain, *DmIh* has a broad pattern of expression [24], presumably also in dopaminergic neurons. To confirm this we performed RT-PCR of isolated dopaminergic cells. Whole brains of flies expressing GFP in dopaminergic cells (see Materials and Methods) were disaggregated and fluorescence-activated cell sorting (FACS) was used to collect GFP expressing cells. As shown in Figure 3, cells that express the TH encoding gene, *ple*, also express *DmIh*.

**Figure 4:**
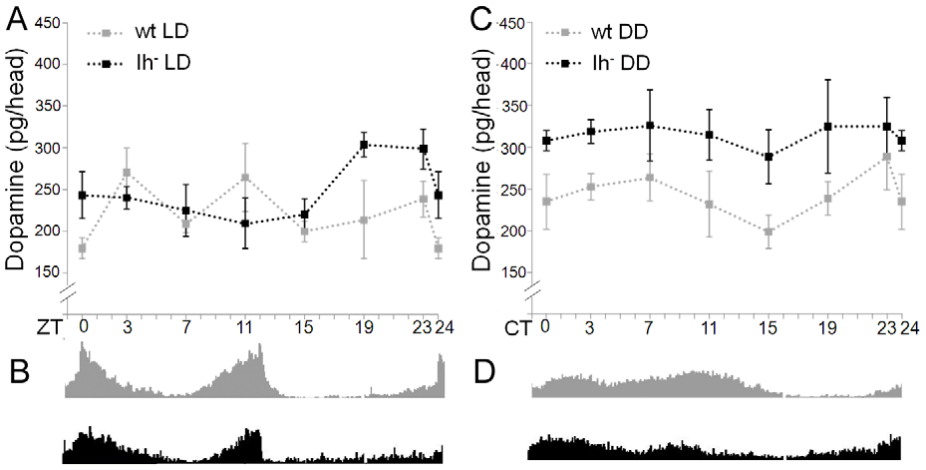
Lack of Ih current alters dopamine levels. Dopamine and activity was measured in 6 to8 days old flies at day 3 or 4 in each condition. Error bars indicate SEM. (A) In LD conditions, *DmIh* mutant flies (black dotted line) lose the characteristic cyclic pattern of dopamine at daytime, and show a notable increase in dopamine levels at night time. To facilitate comparison, dopamine in control flies is displayed with a gray dotted line. (B) Correspondingly, total activity plot of mutant flies (n = 31; bottom black actogram) reveals subtle changes in the circadian locomotor activity when compared with control flies (top gray actogram): activity peak at lights on looks wider and blunted, while peak at lights off ends abruptly. In addition, mutant flies show increased activity during the night compared to control flies. (C) In DD conditions, the level of dopamine in *DmIh* mutant flies (black dotted line) increases drastically throughout the 24 hour period when compared to control flies (gray dotted line). (D) Total daily activity plot of mutant flies (n = 53; bottom black actogram) shows that activity during the second half of the subjective day is reduced compared to control flies (top gray actogram).

Flies deficient for *DmIh* are viable and fertile, allowing us to measure dopamine in adult heads. We first asked whether mutant flies had a normal locomotor activity in LD conditions. Out of 58 flies tested for daily activity during 10 days, 82.7% were rhythmic and 17.3% weakly rhythmic, while 100% of control flies (n = 60) were rhythmic (Table 1). Mean activity patterns in LD also looked similar for control and mutant flies (Figure 4B).

**Table 1:** Rhythmicity parameters.

		%R	%WR	%A		Period	
**LD**	wt (n = 61)	100			(p<0.001)	23.85±0.008	
	Ih^−^ (n = 58)	82.7	17.3			23.86±0.01	
**DD**	wt (n = 47)	76.6	19.2	4.2	(p<0.0001)	23.81±0.05	(p<0.001)
	Ih^−^ (n = 53)	24.53	52.83	22.64		23.26±0.09	

Percentage of control (wt) or *DmIh* mutant (Ih^−^) flies that are rhythmic (%R), weakly rhythmic (%WR), or arrhythmic (%A) in LD or DD conditions. Period (mean±SD) was calculated by chi-square periodogram using rhythmic and weakly rhythmic flies. Statistics refer to difference between control and mutant genotype regarding the percentage of R, WR, and A flies (χ^2^ test), or the period length (Mann-Withney test).

Quantification of dopamine in mutant flies showed that the cyclic behavior of dopamine is lost during daytime (Figure 4A). However, average dopamine amount was not different from control flies (230.9±11.5 for control and 229.5±6.9 for mutant; t_30_ = 0.106; p = 0.916). In contrast, at nighttime mutant flies show a dramatic increase of dopamine (217.4±9.3 for control and 274.3±12.6 for mutant; t_22_ = −3.633; p = 0.001). The different behavior of dopamine oscillation in control and mutant flies was confirmed by a two-way ANOVA, which showed that both genotype and ZT, and the interaction between these two factors, significantly affect dopamine levels (F_13,42_ = 7.755; p<0.001; for details see Table S2). Our results suggest the implication of Ih current in maintaining physiological levels of dopamine, although different mechanisms may be acting in light and dark phases.

### Ih deficient flies tend to lose the characteristic bimodal pattern of activity in presence of light

Dopamine has been implicated in several physiological features, among them arousal and the control of motor function. During the light phase, wild type flies show a bimodal pattern of locomotor activity, peaking at dawn and dusk with lower levels of activity between them (Figure 2B). In DD condition, flies sustain a plateau of activity during the subjective day, instead of the two peaks at dawn and dusk (Figure 2D). Interestingly, dopamine cycling also shows a bimodal pattern at daytime in LD, while during the subjective day in DD it is maintained within a narrow range of values. Although both activity and dopamine levels show a bimodal pattern, the peaks are not coincident. In fact, dopamine peaks at both high and low activity levels, precluding a simple quantitative signal (dopamine)/response (activity) effect (See Discussion). These observations, and the disruption of the dopamine cycling in *DmIh* mutants at daylight, made us wonder if the pattern of diurnal activity would be affected in mutant flies.

**Figure 5:**
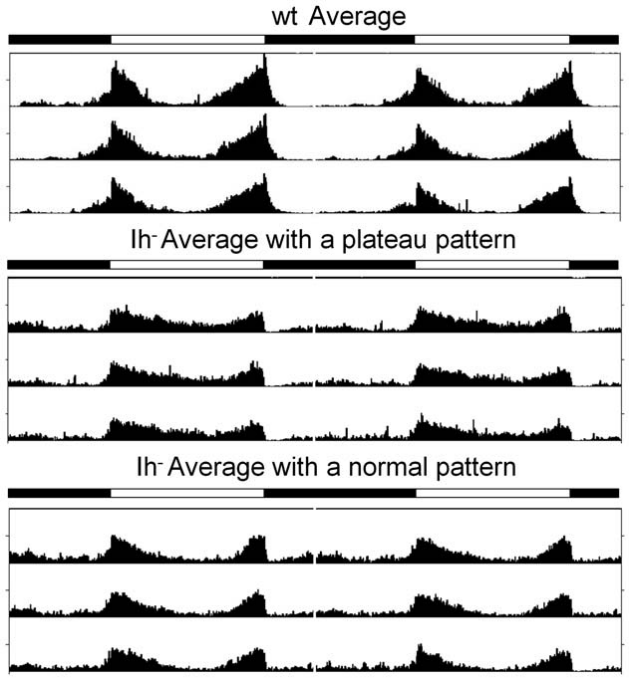
*DmIh* mutant flies lose the characteristic bimodal activity pattern in LD. Average actograms show the typical bimodal pattern of activity in LD in most of the control flies (98.4%, n = 53; top) and in 58.9% of the *DmIh* mutant flies (average of 33 flies; bottom “Ih^−^ Average with a normal pattern”). However, the rest of the *DmIh* mutant flies (41.1%, n = 23) display an altered pattern characterized by a plateau of sustained activity during the light period (middle “Ih^−^ Average with a plateau pattern”). Mutant flies also show a (plateau of?) sustained activity during the night.

When analyzing activity behavior, the mean overall activity of mutant flies is lower (see below), and peak of activity at dawn is blunted (Figure 4B). As mean values in circadian activity analysis may conceal some salient features, we undertook an individual visual inspection of the behavior of each fly and found that about half of *DmIh* mutant flies lack the bimodal pattern of activity (Figure 5), resembling the sustained activity shown by wild type flies during the subjective day in DD (Figure 2D). To get a more objective measure of this behavior, we performed Fourier transformation analysis on control and mutant flies, which provides a quantitative assessment of the rhythmicity at the predominant frequency: normal behavior in LD gives a predominant frequency of 12 hours due to the bimodal rhythmic pattern, while a sustained activity during the light period is expected to yield a predominant frequency of 24 hours. Indeed, while 98.4% of control flies (n = 53) had a predominant frequency of 12 hours, in *DmIh* mutants flies the predominant frequency was 24 hours in 41.1% (n = 56) of the flies. This result suggests an association between the pattern of dopamine oscillation and the pattern of locomotor activity, both being affected by light.

### Lack of Ih current increases dopamine levels in DD

Our results show that in LD conditions the amount of dopamine augments during the night period in *DmIh* mutant flies. Therefore, we measured dopamine levels in *DmIh* mutants in DD conditions to verify if the increase observed at nighttime in LD correlates with a lack of light input whatever the circadian conditions. In constant dark, the proportion of rhythmic *DmIh* mutants drops to 24.5% (Table 1). These flies cannot sustain the characteristic activity plateau at the end of the subjective day (Figure 4D). Interestingly, mutant *DmIh* flies show an increase in dopamine levels in DD similar to that of night in LD (Figure 4C; 244.05±7.0 for the control and 314.98±6.3 for mutant; t_54_ = −7.529; p<0.001). Two-way ANOVA (F_13,42_ = 6.948; p<0.001) showed that in this condition too, genotype and CT significantly affect dopamine levels. However, the pattern of circadian changes is not different between control and mutant flies, because the interaction between these factors was not significant (see Table S2).

Taken together, our results strongly suggest that, in *Drosophila*, Ih current is necessary to prevent an overproduction of dopamine in dark conditions. In addition, we can infer from our results that light input influences cycling of dopamine in an Ih current dependent manner. *DmIh* is expressed in retinal receptors [24], but lack of this current does not prevent light entrainment of circadian rhythms, indicating that mutant flies are not severely impaired for light detection.

### Sleep behavior is altered in *DmIh* mutant flies

**Figure 6:**
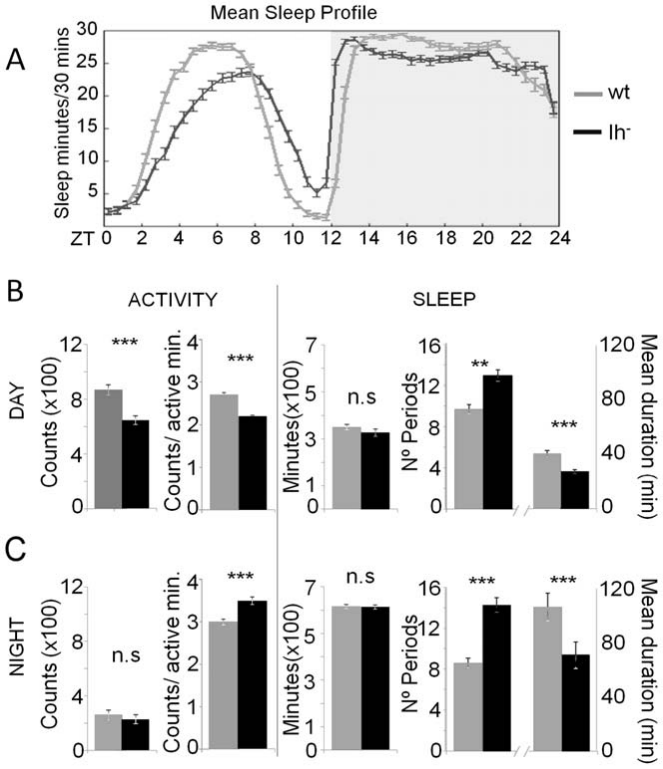
Loss of Ih current affects sleep consolidation. (A) Daily time course (30 min interval) of the amount of sleep in 6 to 8 day-old males of control (n = 64) and *DmIh* mutant (n = 64) genotypes in LD conditions. White and gray areas indicate light and dark periods, respectively. Data points represent mean ± SEM. (B) Rest∶activity parameters of both genotypes in the light period. Mutant flies are hypoactive as infer from the decreased beam crossing counts per active minute. These flies have the same amount of total sleep during the day, but with significantly more sleep bouts of shorter duration. (C) At nighttime total activity of mutant flies is not different from controls, but beam crossing counts per active minute are significantly higher in mutant flies. Total sleep is the same for both genotypes, but, as in the light phase, *DmIh* mutants have more sleep bouts of a shorter duration. Error bars represent two SEMs. Asterisks denote significant differences based on the proper test performed by FlySiesta software (*p<0.05; **p<0.01; ***p<0.001; n.s., not significant).

*Drosophila* has proven to be a good model to study sleep [36], [37], and dopamine has emerged as a key modulator of this physiological state. It has been shown that lengthening the dopamine effect in target neurons, as in flies with a mutation in the dopamine transporter encoding-gene *fumin* (*fmn*), leads to a severe reduction of sleep [26], even though the number of sleep episodes increases considerably [38]. In addition, high dopamine levels in flies lacking the fragile X mental retardation protein (FMR1) increase the number of sleep episodes [39], [40]. Given the anomalous dopamine levels in *DmIh* mutants we expected to see a change in the rest-activity pattern of these flies. Activity was monitored in individual flies in a 12 h light∶dark cycle and rest∶activity parameters were analyzed (Figure 6). Sleep state was defined as bouts of uninterrupted five minutes of inactivity. The non-sleeping periods were considered as waking state.

During the day, total length of the waking period is not different between control and mutant flies. However, a reduction in beam-crossing counts per active minute causes a decrease in the total activity of mutant flies (Figure 6B), suggesting that *DmIh* deficient flies are hypoactive. Surprisingly, at nighttime mutant flies show a significant increase in the counts per active minute when compared with control flies, but total activity is not different (Figure 6C). This is explained by an increase in the non-active waking periods, because control and mutant flies spend the same time in a waking state.

Total sleep is not different between mutant and control flies, both during the day and at night. However, *DmIh* deficient flies show a significant increase in the number of sleep episodes and a decrease in their duration (Figure 6B, C). This tendency toward sleep fragmentation was even more pronounced during the night phase, when absolute dopamine levels are elevated as compared to control flies (Figure 4A). In control flies total sleep is higher at night than at day time due to an inversion of the sleep pattern: the number of sleep episodes is slightly reduced, and their duration is dramatically increased. However, mutant flies do not show this characteristic inversion (Figure 6C). As a result, during the night *DmIh* deficient flies display a significant, considerable increase in the number of episodes and a decrease in their duration when compared with control flies.

**Figure 7:**
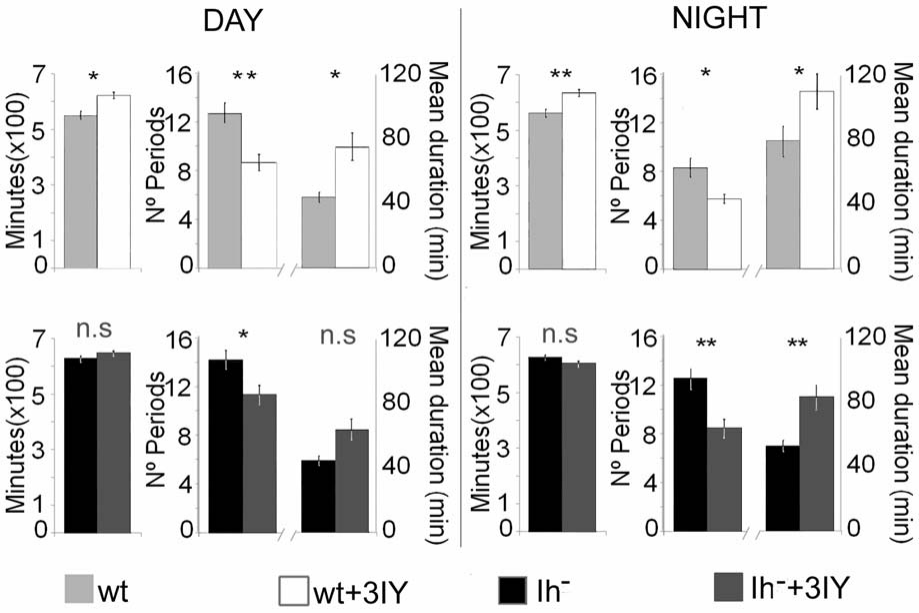
Decreasing dopamine levels by 3IY in *DmIh* null mutant rescues sleep phenotype. Sleep parameters of control and mutants flies under control conditions (flies fed with gelatin solution) or drug treatment (flies fed with 10 mg/ml 3YI in gelatin solution). Drug treatment considerably increases sleep consolidation in control flies (gray vs white bars) both at day and night. In *DmIh* mutant flies, drug treatment rescues the sleep fragmentation phenotype, especially at night time (black vs dark gray bars), when the number of sleep periods and their duration show values similar to non-treated control flies (see text for details). Error bars represent two SEMs. Asterisks denote significant differences based on the proper test performed by FlySiesta software (*p<0.05; **p<0.01; n.s., not significant).

Altering dopamine signaling affects total sleep time and sleep consolidation [41], [42]. Our data show that in *Drosophila*, lack of Ih causes sleep fragmentation without affecting total sleep amount, and the severity of this phenotype seems to relate to high dopamine levels occurring at nighttime. However, it can be argued that this sleep phenotype is not dependent on disruption of dopamine normal fluctuation, but on a dopamine-independent effect caused by the lack of Ih current. To discern between both alternatives, we pharmacologically diminished the amount of dopamine by feeding *DmIh* mutant flies 3-iodotyrosine (3IY), a competitive antagonist of tyrosine hydroxylase (TH) which has been shown to reduce dopamine in flies without producing significant effects on basic behavior and viability [43]. We predicted that if the sleep phenotype of *DmIh* mutant flies, which is basically an increase in sleep fragmentation, is dopamine-dependent, a reduction on dopamine levels would at least partially correct this fragmentation. Indeed, when analyzing the rest∶activity parameters of 3IY-fed flies, it is clear that sleep becomes more consolidated in both control and mutant flies (Figure 7). Moreover, the number (12.9±0.8 and 11.4±0.8 for control and mutant flies respectively at daytime; 8.4±0.8 and 8.5±0.8 for control and mutant flies respectively at nighttime) and duration (in minutes, 46.6±3.4 and 65.7±6.6 for control and mutant flies respectively at daytime; 84.3±10.2 and 87±8.4 for control and mutant flies respectively at nighttime) of sleep episodes of drug treated mutant flies is not significantly different from non-treated control flies (p>0.1), indicating that reducing dopamine levels in *DmIh* mutant flies restores sleep consolidation. Furthermore, drug-treatment has a more pronounced effect at night on mutant flies than on control flies, which is expected based on the higher dopamine content of mutant flies at nighttime. Specifically, drug treatment increases night sleep episode duration by 58% in mutant flies (p<0.001) but only 39% in controls (p<0.05), while episode number is reduced by 33% (p<0.001) and 30% (p<0.05) respectively. Nevertheless, drug treatment increased total sleep only in control flies, suggesting that the effect of dopamine on total sleep time may occur, to some extent, through the modulation of Ih current.

**Figure 8:**
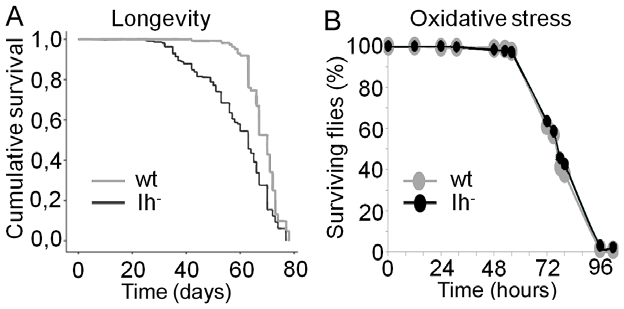
The absence of Ih current shortens lifespan but does not affect resistance to oxidative stress. (A) Lifespan determination of *DmIh* mutant males (black line, n = 234) compared to controls (gray line, n = 145). Survival curves of the two genotypes are significantly different (Mantel-Cox Statistic = 32.281, df = 1, p<0.0001). (B) Resistance to oxidative stress of *DmIh* mutant males (black line, n = 511) is not different from the controls (gray line, n = 384).

Sleep fragmentation is characteristic of aging and has been proposed to be related with a concomitant buildup of oxidative damage [44]. In laboratory conditions (25°C and 60% humidity) *Drosophila* flies live more than two months and the effect of age in sleep fragmentation is not perceptible until the fourth week. Our sleep∶activity recordings were always performed in 6 to 8 days old flies, precluding an effect of chronological age in our results. In order to see if *DmIh* mutant flies might be considered physiologically elderly, we performed a test of longevity. The survival curves of *DmIh* mutant male flies reared at 25°C did not show a reduction in the maximum life-span and only a slight reduction in the median (10%), but the death rate during the exponential phase was increased (Figure 8A). It has been reported that resistance to oxidative stress declines with age [45]. To test the possibility that *DmIh* null flies might have a precocious senescence, based in their augmented death rate in the second month of their life, we analyzed their survival when exposed to a source of hydrogen peroxide. Mutant flies were not more susceptible to oxidative stress compared with the control line (Figure 8B). This result rules out the possibility of an increased buildup of oxidative damage as a determining factor of the increased death rate of *DmIh* mutant flies. Interestingly, *apathetic*, a spontaneous mouse mutation in HCN2, a homologue of *DmIh* in vertebrates, also shows an increased mortality, although the cause of death has not been determined [46].

### *DmIh* mutant flies show a modified activity rhythm in free running

**Figure 9:**
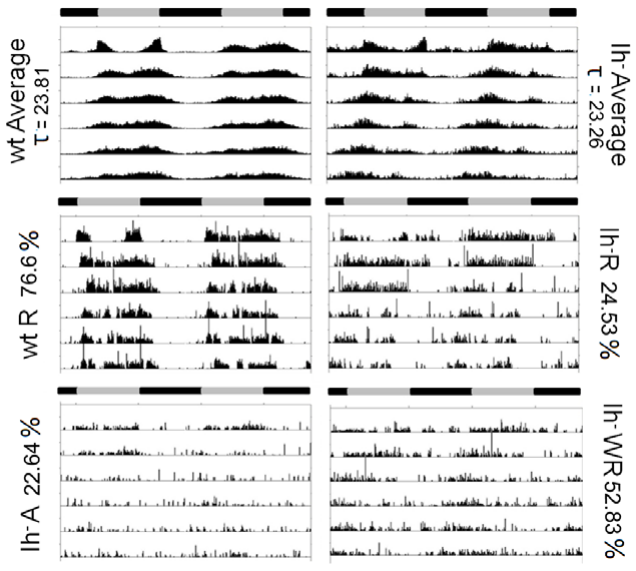
In constant dark, *DmIh* mutant flies shorten the circadian period. Average (top) and single fly representative (middle and bottom) double-plotted actograms of control and *DmIh* mutant flies in DD conditions. Flies were entrained for three days in LD conditions prior to being released to DD. The first day of each actogram corresponds to the last day in LD. Mutant flies included rhythmic flies (Ih-R), weakly rhythmic flies (Ih-WR), and arrhythmic flies (Ih-A) (see Table 1 for circadian parameters). Rhythmic mutant flies display a shorter period and a failure to maintain the activity plateau during the subjective day.

**Figure 10:**
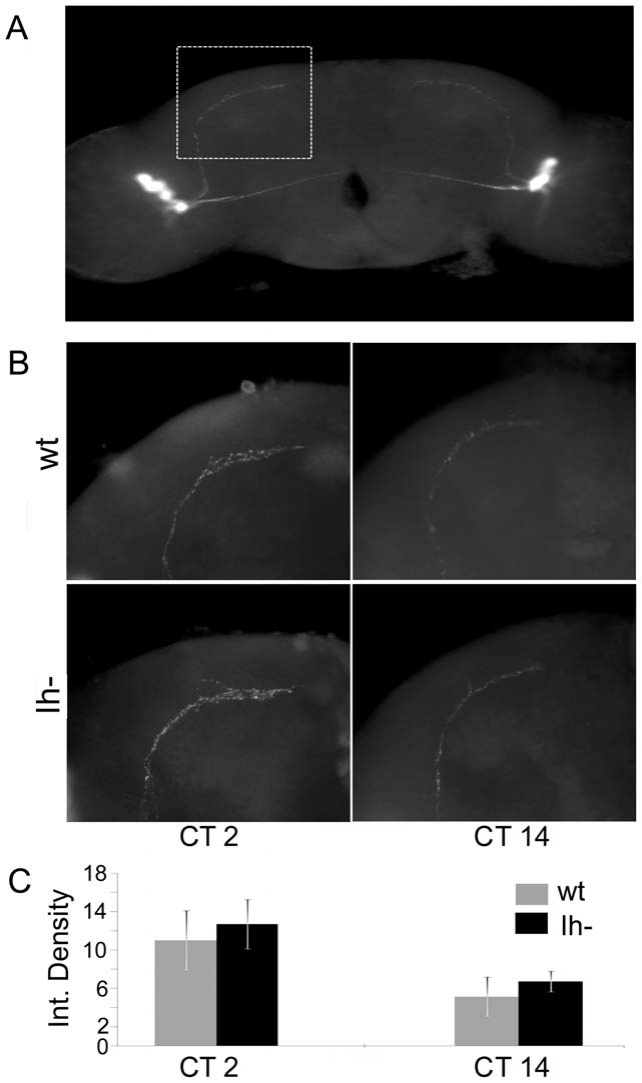
Lack of *Ih* current does not disrupt PDF clock output. *Ih* null mutants show normal circadian release of pigment dispersing factor (PDF) in DD conditions. (A) Anti-PDF staining of brains showing the LNv cells and their dorsal projections. Dotted line shows the area selected for analyses. (B) PDF immunostaining in the dorsal projections shows normal oscillation of this factor in wild type (wt) and *Ih* null mutants (*Ih* ^−^), with high levels in the early day (CT2) and low levels in the early night (CT14). (C) Quantification of PDF immunoflorescence (Integrated Density) in wt and *Ih* ^−^ at CT2 and CT14 (Error bars indicate standard deviation) There are no differences between genotypes at any time point (t_13_ = −1.121, p = 0.283 for CT2; t_11_ = −1.794, p = 0.100 for CT14).

When *DmIh* mutant flies are released to DD conditions, to unravel the endogenous clock, the activity rhythm is different from control flies (Figure 9). Most remarkably, 22.6% of the mutant flies become arrhythmic and only 24.5% show robust rhythmicity (Figure 9, Table 1). When analyzing activity patterns in those rhythmic *DmIh* deficient flies, the most striking fact is a shortening of period length (23.3 h, Table 1). Moreover, analyzing the average actogram (n = 13; Figure 9), it becomes evident that activity decays in the second half of the subjective day. These anomalous behaviors explain the shape of the average daily activity plot of mutant flies shown in Figure 4D, which shows an early decay of activity during the subjective day. We asked if the lack of Ih current in the circuitry underlying the final output of the clock may account for this anomalous behavior. As an assay of clock output we looked at the pattern of PDF release from dorsal projections of the master clock neurons (LNvs, ventral lateral neurons), which has been shown to vary in a circadian manner [47]. As shown in Figure 10, PDF secretion from LNvs in *DmIh* mutant flies does not differ from control flies, suggesting that the modification of their circadian rhythm may be due to factors downstream of the LNv clock, or alterations in other, non-PDF clock cells [48].

## Discussion

Here, we show how dopamine levels in adult *Drosophila* flies cycle in a circadian manner and provide important evidence for a role of Ih current in this phenomenon. Moreover, we provide evidence that Ih current is necessary to consolidate sleep. As HCN channels, responsible for Ih current in mammals, are becoming pharmacological targets for cardiac diseases, foreseeing the consequences of abolishing Ih current is a requirement for searching new therapies.

Even though our data on dopamine levels was obtained from whole heads, various reasons make us confident that dopamine oscillation precisely reflects the behavior of neural dopamine. First, contribution of hypodermic dopamine to whole head dopamine levels has been reported to be no more than 15% [28]. Second, *pale* transcripts, which encode tyrosine hydroxylase (TH), have been shown to cycle circadianly in whole heads but not in bodies [31], indicating that hypodermal dopamine is not under circadian control. Finally, expression of *Ih* in adult heads has been detected in neural tissues [24], suggesting that Ih affects neural dopamine. Our attempts to measure dopamine in brains have shown that time spent at dissecting is critical, as dopamine becomes rapidly oxidized and dopamine values drop to undetectable values for dissecting times over five minutes (not shown). Moreover, because flies used for each time point must be kept on ice while waiting to be dissected, these anesthetizing conditions imply a change in their physiological state that could influence dopamine content, possibly differentially affecting mutant and wild type flies, and thus precluding the use of this technique for obtaining reliable dopamine measures.

Daily fluctuations of dopamine have already been reported in mammals, and also suggested in *Drosophila* based on the circadian oscillations of TH and dopamine receptor responsiveness, but a detailed study of dopamine levels at different time points was lacking. Our results show rhythmicity in the daily levels of dopamine in *Drosophila*, with a pronounced bimodal pattern at daylight and a smooth increment towards the end of the night. When analyzing the contribution of the light signal versus the circadian control in this rhythmic behavior, through a comparison of LD and DD dopamine values, it turns out that the bimodal pattern during the day is driven by light while dopamine levels at night are under circadian control. A link between light and dopamine is supported by studies in vertebrates showing that dopamine plays critical roles in the light-induced resetting of the peripheral retinal circadian clock [49], [50], but also by a recent report in *Drosophila* demonstrating that dopamine is necessary for low light circadian entrainment [34]. The circadian control of dopamine levels at night may reflect the importance of keeping adequate dopamine signaling. It has been shown that the *Drosophila* dopamine receptor dDA1 responds homeostatically by downregulating its expression when dopamine signaling is increased [38]. The physiological role of dropping the levels of dopamine just before the onset of the light phase could be a means of resetting proper amounts of receptors to respond adequately to the changing dopamine levels during the light phase.

How light affects the dopamine outcome is not known, but our results clearly demonstrate the implication of Ih current in maintaining its bimodal pattern. *DmIh* is expressed in photoreceptors [24] and although lack of this current does not impair gross responses to light, we cannot rule out an effect on the circuitry conveying light reception to inner brain centers. In fact, evidence for an involvement of Ih in early visual processing comes from the side effects reported, in dim light or darkness, by cardiac patients treated with HCN inhibitors [51] and by investigations that suggest that Ih current in the rod retinal pathway may contribute to shape the retina light response [52], [53].

Ih current is also involved in maintaining physiological levels of dopamine, because abolishing this current strongly increases dopamine amount in dark conditions. A number of studies have implicated the hyperpolarization-induced, nonselective cation conductance Ih in the firing activity of mammalian dopaminergic neurons, emerging as one determinant of their spontaneous firing rate [8], [9]. Our result could be surprising in view of the results on dissociated dopaminergic neurons. When pharmacologically blocking this current, cultured dopaminergic neurons lower their firing rate, presumably leading to reduced dopamine release, an outcome apparently contrary to our results. However, the contribution of Ih current to the final performance of a neuron is not so straightforward. Despite the fact that Ih provides a depolarizing current at sub-threshold potentials, results from several studies have indicated that it has a paradoxical inhibitory effect by activating the Im potassium current [54]. Also, the different subcellular localization of the channel endows different electrical properties to the neuron [55]. Moreover, *DmIh* mutant flies lack Ih current not only in dopaminergic neurons, but in the entire nervous system, possibly affecting input signals to these cells. Finally, dopamine autoreceptors provide important feedback control during dopamine signaling by governing firing rate, synthesis, and release. Therefore, by acting at different cellular and subcellular locations, Ih current could be critical for proper functioning of the dopaminergic negative feedback loop to prevent excessive release of neurotransmitter.

One of the consequences of the anomalous dopamine amount, when abolishing Ih current, is sleep fragmentation. Previous reports have shown that reducing dopamine signaling, through mutations in dopamine receptors, increases the duration of sleep episodes, suggesting more consolidated sleep [38], [56]. Conversely, enhanced dopamine signaling is associated with sleep fragmentation [26]. *DmIh* deficient flies show a dramatic fragmentation of sleep at nighttime, which is consistent with their increased dopamine levels. This trend towards sleep fragmentation is also observed in mutant flies at daytime, suggesting that disrupting dopamine cycling during the light period can also affect sleep consolidation. Results showing that pharmacologically decreasing the amount of dopamine restores sleep consolidation in mutant flies are consistent with this phenotype being dopamine-dependent (Figure 7). Our evidence suggests that Ih current, possibly through maintaining proper levels of dopamine, have an effect on the consolidation of sleep.

In general, genetic and pharmacological changes in dopamine content affect both total sleep and sleep consolidation in flies [26], [38], [42], and that is what we observe in control flies. Surprisingly, *DmIh* flies have elevated dopamine levels and sleep fragmentation, but total sleep is not significantly altered, nor even by 3YI-treatment. Because the lack of Ih current is the basic difference between control and mutant flies, their differential influence on total sleep must rely on the Ih current itself, or on its possible effects on the release of other neuromodulators involved in the regulation of sleep [56]. It would be interesting to tackle this issue in future investigations.

A number of reports positively correlate dopamine and locomotor activity [26], [57]–[59]. Our data showing that when the bimodal pattern of dopamine is lost (in *DmIh* mutant flies), more than 50% of the flies also lack the bimodal activity pattern (Figure 5), are consistent with an association between dopamine oscillations and locomotor activity. Nevertheless, caution should be taken when interpreting these results because each dopamine measure is an average of 20 brains from a mixture of flies displaying the two different locomotor patterns, i.e. bimodal and non-bimodal. Nevertheless, dopamine should be considered as a modulator of activity rather than responsible for a quantitative signal/response effect. In fact, transient activation of TH-expressing dopaminergic cells (using transgenic ion channels) has opposite effects on activity depending on the previous behavioral state right before photostimulation [60]. This could explain the variability found in the locomotor activity pattern of flies (Figure 5 and 9), as well as why the bimodal patterns in LD of both dopamine and activity are not exactly coincident (Figure 2A, B). Nevertheless, dopamine signalling has also been involved in many other behavioral processes, such as courtship, visual, olfactory and appetitive learning, or mechanosensation [61]. The emerging picture indicates that sleep/activity, behavioral arousal, and even learning and memory, are influenced by anatomically distinct sets of dopaminergic cells. Moreover, besides sleep/activity, many of these behaviors show circadian patterns, with maximum performance usually attained during the (subjective) night [62]–[66]. Therefore, variations of dopamine levels may differ at different anatomical localizations, complicating an interpretation aimed at explaining an individual behavior in terms of total dopamine levels. Even so, it is tempting to speculate that the cyclic pattern of total dopamine in DD conditions may reflect dopamine requirements for both wakefulness (maximum during the subjective day) and behavioral arousal (maximum during the subjective night), but further experiments would be needed to established such a relationship.

The locomotor pattern of activity in DD reveals some interesting features of *DmIh* mutant flies: 22.6% become arrhythmic and only 24.5% show robust rhythmicity, and when analyzing the circadian parameters of those rhythmic flies, the most striking fact is a shortening of period length. These abnormalities of circadian rhythm could be a consequence of lack of Ih current in the circuitry underlying the final output of the clock, but PDF secretion from LNvs in *DmIh* mutant flies does not differ from control flies, suggesting that altered circadian rhythm may be due to factors downstream of the LNv clock, or alterations in other, non-PDF clock cells [48]. Further support for normal activity of the LNv morning-oscillator comes from the observation that, contrary to wild type flies, *DmIh* mutant flies kept in DD conditions maintain activity in the first half of the subjective day (Figure 9), which is under LNv control [67]. On the contrary, while wild type flies tend to be more active during the subjective evening, mutant flies show a progressive decay of activity during this period, suggesting alterations in the clock circuit responsible for this phenomenon.

Interestingly, similar defects in rhythmicity and period length have been reported for *ebony* mutants, and also the central LNv clock seems not to be affected [68]. These mutant flies lack N-β-alanyl-dopamine synthetase activity in glial cells, and have elevated levels of dopamine. Although we cannot assume that the abnormalities of circadian rhythm in *DmIh* mutant flies are due to elevated dopamine levels, mutual interactions between dopamine and peripheral circadian clocks have been reported in other systems. In the vertebrate retina, dopamine regulates the phase and amplitude of retinal molecular rhythms and participates in light-induced resetting [49], [69]. In rodents, activation of the dopamine D2 receptor signaling cascade results in enhancement of clock genes transcription [68], and disruption of dopamine signaling leads to disruption of circadian rhythms in selected forebrains regions and consequent alterations of circadian locomotor behavior [70], with no effect on molecular rhythms in the central suprachiasmatic nucleus (SCN). Moreover, numerous studies have revealed the existence of a methamphetamine-sensitive circadian oscillator, further supporting a role for the mesolimbic-dopaminergic system as a SCN-independent oscillator [71]. Thus, dopamine emerges as an important regulator of peripheral brain clocks in vertebrates, a role that may well be conserved in *Drosophila*.

A link between Ih current and dopamine signaling has been suggested by electrophysiological studies in mammalian dopaminergic cells, and by isolated reports involving Ih current in various dopamine-related disorders [18]–[22]. However, to our knowledge, our work represents the first *in vivo* analysis in which this association has been demonstrated and the behavioral consequences have been analyzed in a complete organism, and, interestingly, our results suggest significant evolutionary conservation. Moreover, we have demonstrated that Ih current regulates dopamine circadian and light-dependent oscillations, and provided evidences indicating that cyclic dopamine signaling is essential for normal behavior. Therefore, our data should be considered not only in view of the value of HCN channels as therapeutic targets, but also when approaching functional and pathological studies of dopamine-related processes. In this sense, our data corroborate the usefulness of *Drosophila* as a model for these types of studies.

## Materials and Methods

### Fly strains

Flies were grown at 25°C under a 12 h∶12 h light cycle in standard *Drosophila* food. Fly strains used were Canton-S, *w^1118^;sna^Sco^/SM6a*, *w^1118^;TH Gal4, w^1118^;UAS:GFP* and *y^1^ w^1118^ hspFLP;sna^Sco^/SM6a* (Bloomington Stock Center, Indiana) and *w^1118^;pBac^{RB}Ihe01599^*, and *w^1118^;pBac^{WH}Ihf01485^*(Exelixis Collection, Harvard). *DmIh* mutant flies were obtained by deleting the S1–S3 core domain upon recombination of the two pBac elements. Recombination was induced following described protocols [27]. Briefly, daily 1 hour-heat shocks (37°C) were given to the progeny from the cross *y^1^ w^1118^ hspFLP*; *pBac^{RB}Ihe01599^/SM6a*×*y^1^ w^1118^ hspFLP;pBac^{WH}Ihf01485^/SM6a*, starting at the second day after the cross was established. Heat-shocked males of the genotype *hspFLP*; *pBac^{RB}Ihe01599^/pBac^{WH}Ihf01485^* were crossed to *w^1118^;sna^Sco^/SM6a* females, and individual lines were established from male descendants and checked by PCR to determine if recombination had occurred. Two *DmIh*-deficient lines were identified, and one of them was backcrossed into a Canton-S (CS) background for five generations to establish one isogenic w^+^ control (*wt*) and one isogenic w^+^ mutant (*DmIh*) line, which were used in all experiments.

### Molecular characterization of the *DmIh* mutant allele

Genomic DNA was isolated from 10 control and recombinant flies with High Pure PCR Template Preparation Kit (Roche), and used for PCR amplification. Primers AACGATGATCTGAGCACACG and CAGCGTTGTCTTGTTGCATAA were used for amplification of the *DmIh* core region. A single primer TGCATTTGCCTTTCGCCTTAT was used as both forward and reverse primer for amplification of the newly formed pBac element with the Taq Long Extent (Roche). PCR products were analyzed in 0.8% agarose gels.

### Dopamine quantification and statistics

For HPLC analysis, 10 heads of 6 to 8 day-old males, entrained for 3 to 4 days in LD or DD conditions, were homogenized in 100 µl of ice-cold 0.1 M perchloric acid. The samples were centrifuged at 13.000 rpm for 10 min and supernatant filtered through a 0.45 µm PVDF centrifuge filter (Teknokroma). Dopamine levels were measured with a Varian 1200L HPLC with triple quadrupole LC/MS equipped with a Zorbax SB C18, 5 µm, 4.6 mm×150 mm column (Agilent).

Each ZT and CT point represents the mean value of two independent experiments, with duplicated HPLC measures. HPLC measurements coefficient of variation were 8.04% and the inter-experiment coefficient of variation was 7.97%. All statistical evaluations were performed with SPSS13 (SPSS, Chicago, IL, USA). Because dopamine measurements fulfilled the assumptions of the normal distribution and the homogeneity of variance, data were compared with one or two way ANOVA followed by the Bonferroni's post hoc test. Significant interactions in the ANOVAs were followed up with *t* tests.

### Isolation of dopaminergic neurons by fluorescence activated cell sorting (FACS) and RT-PCR analysis

In order to determine whether dopamine cells express the *DmIh* gene, we performed RT-PCR assays in isolated *ple* (TH) expressing cells by Fluorescence Activated Cell Sorting (FACS). 50 *THG4/UAS:GFP* adult brains were dissected and mechanically dissociated in Trypsin [72]. Following dissociation, 1% FBT (fetal bovine serum) was added and cells were pelleted at low speed and resuspended in 1% FBT. Cells were subjected to FACS using a FACSVantage SE cytometer, yielding 623 GFP-positive cells. RNA was isolated from the GFP-positive and a similar number of GFP-negative cells using RNAspin Mini (GE Healthcare). Reverse transcription and PCR reaction was performed (in one-step protocol) with the Ready-To-Go RT-PCR Beads (GE Healthcare). Primers used were 5′-AACGATGATCTGAGCACACG-3′ and 5′-CAGCGTTGTCTTGTTGCATAA-3′ for *Ih* amplification, and 5′-CGCAGCAAGGCAAATGAT-3′ and 5′-AGGAGATGCCCTCCTTGAG-3′ for the neural isoform of the *ple* gene.

### Activity and sleep assays

Two to three day-old males were placed in 65 mm×3.5 mm glass tubes (Trikinetics, Waltham, MA) containing standard *Drosophila* food. Locomotor activity was collected with *Drosophila* Activity Monitoring Systems (DAMS, Trikinetics) in 1 min bins, and circadian data were analyzed using Clocklab (Actimetrics Software). For LD analysis, flies were kept in 12 h∶12 h light-dark cycles for at least 6 days. For DD analysis, flies were first entrained in 12 h∶12 h LD cycles during 3 days, and activity data were analyzed for 6 days. To assess rhythmicity, data were analyzed by chi-square periodogram. Flies that showed clear rhythms through actogram analysis and a well-defined peak close to 24 h in the periodogram were classified as rhythmic and included in the calculation of average period. Flies that failed to yield a significant period in the periodogram analysis and showed random distribution of activity in actograms were classified as arrhythmic. Those that showed recognizable, but weak rhythms in actograms, and resulted in periodograms that displayed insignificant or barely significant periods, were considered weakly rhythmic. To analyze the bimodal vs. unimodal pattern of locomotor activity in LD, data from 6 consecutive days were analyzed by Fourier transformation to yield the predominant frequency. In all cases, at least two independent experiments were analyzed.

For rest∶activity analysis, flies were acclimated in behavior tubes at 25°C in 12 h light∶12 h dark (LD) conditions for 3 to 4 days, and data were collected for the following 2 consecutive days. Active Sleep was defined as any period of uninterrupted behavioral immobility lasting more than 5 min. Waking state was any non-sleeping state, and an active minute was defined when ≥1 count/min was recorded. DAMS data were analyzed with FlySiesta custom-designed MATLAB software [73]. Sleep and activity parameters were analyzed for each 12 hr period of LD and averaged over 2 days for each condition.

### Drug treatment

Pharmacological treatment with 3-iodotyrosine (3IY) (Sigma) was carried out as described [43]. Briefly, three to four day-old males were placed in 65 mm×3.5 mm glass tubes (Trikinetics, Waltham, MA) containing 8% gelatin, 5% sucrose, and 10 mg/ml 3IY.

### Lifespan analysis

Males of genotypes under study were collected within 24 hr of eclosion under brief CO_2_ anesthesia and housed in groups of 25. They were raised at 25°C under a 12 h∶12 h light cycle and transferred to fresh food vials every 2–3 days. Dead flies were scored daily.

### Oxidative stress

Three day-old males were transferred to vials with filter papers soaked with 1 ml of a 10% sucrose solution with or without 1% H_2_O_2_ for the first 24 h. In subsequent days 5% H_2_O_2_ was used. Dead flies were scored every 8 h.

### Immunohistochemical assays

*Drosophila* adults were entrained for three days in LD and were dissected in the third day in DD at CT2 and CT14. Brains were fixed in 4% Formaldehyde solution. The primary antibody used was mouse anti-PDF (JH Park, University of Tennessee) and was detected with anti-mouse antibody conjugated with Rhodamine-RedX (Jackson ImmunoResearch, PA, US). Images were acquired under the same parameters using a Leica Wild MPS52 microscope and analyzed with ImageJ software. Eight hemispheres were measured for each genotype and condition.

## Supporting Information

Table S1 Comparison of Dopamine cycling in LD and DD conditions in control flies. Two way ANOVA on dopamine datapoints was performed to assess the effect of circadian time (ZT/CT) and light condition (LD or DD). A two-way ANOVA including both factors and their interaction is significant (F_13,42_ = 4.760; p<0.001). Both factors have a significant effect, and also their interaction, meaning that the dopamine cycling along the 24 h period is different in LD and DD.
(DOC)

Table S2 Effect of *DmIh* mutation on Dopamine cycling in LD and DD conditions. Two way ANOVA on dopamine datapoints was performed for both conditions (LD and DD) to assess the effect of genotype (control or *DmIh* mutant) and circadian time (ZT/CT). In L∶D, both genotype and ZT significantly affect dopamine levels. Dopamine levels vary with ZT in both genotypes, but the changes along the day are different in control and *DmIh* mutant flies, given that the interaction between Genotype and ZT is significant. In D∶D, genotype and CT significantly affect dopamine levels. However, the daily changes are not different between control and *DmIh* mutant flies, because the interaction between these factors is not significant.
(DOC)
