# Supplementary material for: Ih Current Is Necessary to Maintain Normal Dopamine Fluctuations and Sleep Consolidation in Drosophila
Source: PLoS One. 2012 May 4;7(5):e36477. doi: 10.1371/journal.pone.0036477 (PMC3344876; doi:10.1371/journal.pone.0036477)
Supplement: Table S1 — Comparison of Dopamine cycling in LD and DD conditions in control flies. Two way ANOVA on dopamine datapoints was performed to assess the effect of circadian time (ZT/CT) and light condition (LD or DD). A two-way ANOVA including both factors and their interaction is significant (F13,42 = 4.760; p<0.001). Both factors have a significant effect, and also their interaction, meaning that the dopamine cycling along the 24 h period is different in LD and DD. (DOC) [file pone.0036477.s001.doc]

**Table S1. Comparison of dopamine cycling in LD and DD conditions in control flies.**

Two-way ANOVA on dopamine data points was performed to assess the effect of circadian time (ZT/CT) and light condition (LD or DD).

A two-way ANOVA including both factors and their interaction is significant (F_13,42_=4.760; p<0.001). Both factors have a significant effect, and also their interaction, meaning that the dopamine cycling along the 24h period is different in LD and DD.

| **Source of variability** | **d.f.** | **MS** | **F** | **p** |
| --- | --- | --- | --- | --- |
| LD-DD | 1 | 50.104 | 5.985 | 0.019 |
| ZT/CT | 6 | 51.133 | 6.108 | <0.001 |
| LD-DD x ZT/CT | 6 | 26.854 | 3.208 | 0.011 |
| Error | 42 | 8.371 |  |  |
|  | R^2^= 0.596 | | | |
