# Supplementary material for: Ih Current Is Necessary to Maintain Normal Dopamine Fluctuations and Sleep Consolidation in Drosophila
Source: PLoS One. 2012 May 4;7(5):e36477. doi: 10.1371/journal.pone.0036477 (PMC3344876; doi:10.1371/journal.pone.0036477)
Supplement: Table S2 — Effect of DmIh mutation on Dopamine cycling in LD and DD conditions. Two way ANOVA on dopamine datapoints was performed for both conditions (LD and DD) to assess the effect of genotype (control or DmIh mutant) and circadian time (ZT/CT). In L∶D, both genotype and ZT significantly affect dopamine levels. Dopamine levels vary with ZT in both genotypes, but the changes along the day are different in control and DmIh mutant flies, given that the interaction between Genotype and ZT is significant. In D∶D, genotype and CT significantly affect dopamine levels. However, the daily changes are not different between control and DmIh mutant flies, because the interaction between these factors is not significant. (DOC) [file pone.0036477.s002.doc]

**Table S2. Effect of *DmIh* mutation on dopamine cycling in LD and DD conditions.**

Two-way ANOVA on dopamine data points was performed for both conditions (LD and DD) to assess the effect of genotype (control or *DmIh* mutant) and circadian time (ZT/ CT).

In LD, both genotype and ZT significantly affect dopamine levels. Dopamine levels vary with ZT in both genotypes, but the changes along the day are different in control and *DmIh* mutant flies, given that the interaction between Genotype and ZT is significant.

In DD, genotype and CT significantly affect dopamine levels. However, the daily changes are not different between control and *DmIh* mutant flies, because the interaction between these factors is not significant.

|  | **LD** | | | |  | **DD** | | | |
| --- | --- | --- | --- | --- | --- | --- | --- | --- | --- |
| **Source** | **d.f.** | **MS** | **F** | **p** |  | **d.f.** | **MS** | **F** | **p** |
| Genotype | 1 | 77.574 | 11.250 | 0.002 |  | 1 | 704.392 | 67.764 | <0.001 |
| ZT/CT | 6 | 47.867 | 6.942 | <0.001 |  | 6 | 32.261 | 3.104 | 0.013 |
| Genotype x ZT/CT | 6 | 55.056 | 7.985 | <0.001 |  | 6 | 6.822 | 0.656 | 0.685 |
| Error | 42 | 6.895 |  |  |  | 42 | 10.395 |  |  |
|  | R^2^= 0.706 | | | | R^2^= 0.683 | | | | |
